# Supplementary material for: Survivorship Bias Explains Age-Dependent Extinction in Fossil Genera
Source: bioRxiv. 2026 Jul 24:2026.07.21.739780. Preprint. [Version 1] doi: 10.64898/2026.07.21.739780 (PMC13419402; doi:10.64898/2026.07.21.739780)
Supplement: 1 [file NIHPP2026.07.21.739780v1-supplement-1.pdf]

bioRxiv preprint doi: https://doi.org/10.64898/2026.07.21.739780; this version posted July 24, 2026. The copyright holder for this preprint (which was not certified by peer review) is the author/funder, who has granted bioRxiv a license to display the preprint in perpetuity. It is made available under a CC-BY 4.0 International license.

SUPPLEMENTARY INFORMATION

Appendix A: Direct comparisons of narrowly defined sister cohorts are statistically noisy

The most direct test of survivorship bias would compare genera originating immediately before and immediately after an earlier filtering event A, and ask how well those two cohorts survive a later focal event B. In this approach, each cohort is restricted to genera originating in a single interval adjacent to A and still alive immediately before B. However, as we will see, the small size of these cohorts limits the utility of this approach.

To see this, let's use these cohorts to test a much simpler hypothesis: that older genera survive better than younger genera. This broad age dependence is well established and clearly visible in the aggregate event survival curves of the main text. If the direct sister-cohort comparisons struggle to recover even this strong effect, they are unlikely to have sufficient power to detect the considerably subtler signal sought in this work.

Fig. S1 applies the direct sister-cohort comparison to every ordered pair of extinction events. For each pair, it compares the survival probabilities of the two cohorts (survive till B and originating just before A, versus survive till B and originating just after A). Cell color indicates the posterior evidence for the sign of

$$\Delta = P(\text{survive } B \mid \text{before } A) - P(\text{survive } B \mid \text{after } A),$$

with red supporting greater survival of the older cohort, blue supporting the reverse, pale colors indicating weak evidence, and gray indicating that one or both conditioned cohorts are empty.

Overall, the matrix is reassuringly biased toward red, consistent with the well-established tendency of older genera to survive better. However, many comparisons are undefined, very few individual comparisons provide strong evidence, and those that do are concentrated near the diagonal, where the two cohorts have experienced relatively little attrition before the focal event. Thus, even the broad age-dependence visible in the aggregate event survival curves is only weakly recovered by direct sister-cohort comparisons. Establishing the much subtler signal sought in this work—that filtering events produce survival differences beyond those expected from ordinary age dependence—would therefore be even more challenging. This motivates the nested retrospective cohorts used in the main text.

Appendix B: Discontinuities in event survival curves are not explained by cohort age alone

Event survival curves in Fig. 1 show pronounced discontinuities near some earlier extinction events. One possible explanation is purely compositional: a major extinction may sharply alter which genera remain in the retrospective cohort, producing a corresponding change in the cohort's mean age.

Supplementary Fig. S2 evaluates this possibility by plotting the mean genus age at the focal event for the same retrospective cohorts used to construct the three example survival curves in Fig. 1. The mean-age curves change much more smoothly than the corresponding event survival curves, including across major earlier extinction events. Thus, the discontinuities in Fig. 1 cannot be explained solely by changes in cohort age.

Appendix C: Refined parameter search around the optimum

Figure S3 shows a local refinement of the parameter search around the minimum identified by the coarse grid in Fig. 4. At this finer resolution, the objective function exhibits substantial point-to-point variation arising from the finite number of stochastic model replicates.

Although the refined search does not isolate a unique best-fitting point within the low-error basin, it shows that all near-optimal solutions lie very close to zero toughness growth ($|\lambda_{\text{opt}}| \lesssim 0.001$). As explained in the main text, a deterministic toughness change comparable in magnitude to the event-specific stochastic variation represented by the luck factor would require $\lambda \approx 0.26$. Thus, Figure S3 shows that any deterministic change in intrinsic toughness over a genus lifetime is negligible compared to the stochastic variation represented by the luck factor.

Appendix D: Calibration of the event-weirdness statistic

Before interpreting empirical event-weirdness scores, it is important to verify that the statistic behaves as expected under the fitted null model. Supplementary Fig. S4 applies the complete event-weirdness calculation (see Methods and main text Fig. 5) to synthetic fossil records generated by the fitted toughness-plus-luck model. The median synthetic weirdness remains close to zero, with no extinction event receiving a consistently elevated or depressed weirdness score. This demonstrates that the event-weirdness statistic is well calibrated under the fitted null model.

bioRxiv preprint doi: https://doi.org/10.64898/2026.07.21.739780; this version posted July 24, 2026. The copyright holder for this preprint (which was not certified by peer review) is the author/funder, who has granted bioRxiv a license to display the preprint in perpetuity. It is made available under a CC-BY 4.0 International license.

Appendix E: Independence of event weirdness from extinction magnitude

The event weirdness score introduced in Fig. 5 is intended to quantify *which* genera are eliminated during an extinction event, conditional on the observed number of extinctions. It is therefore designed to assess the composition of the extinction list rather than the overall severity of the event. To verify this property, Supplementary Fig. S5 plots event weirdness against extinction fraction for both the empirical Sepkoski record and synthetic fossil records generated by the fitted toughness-plus-luck model. As expected, the correlation between event weirdness and extinction fraction is weak. Panel A highlights the four events flagged in the main text, confirming that the unusually structured events flagged by this analysis are not simply the largest extinction events.

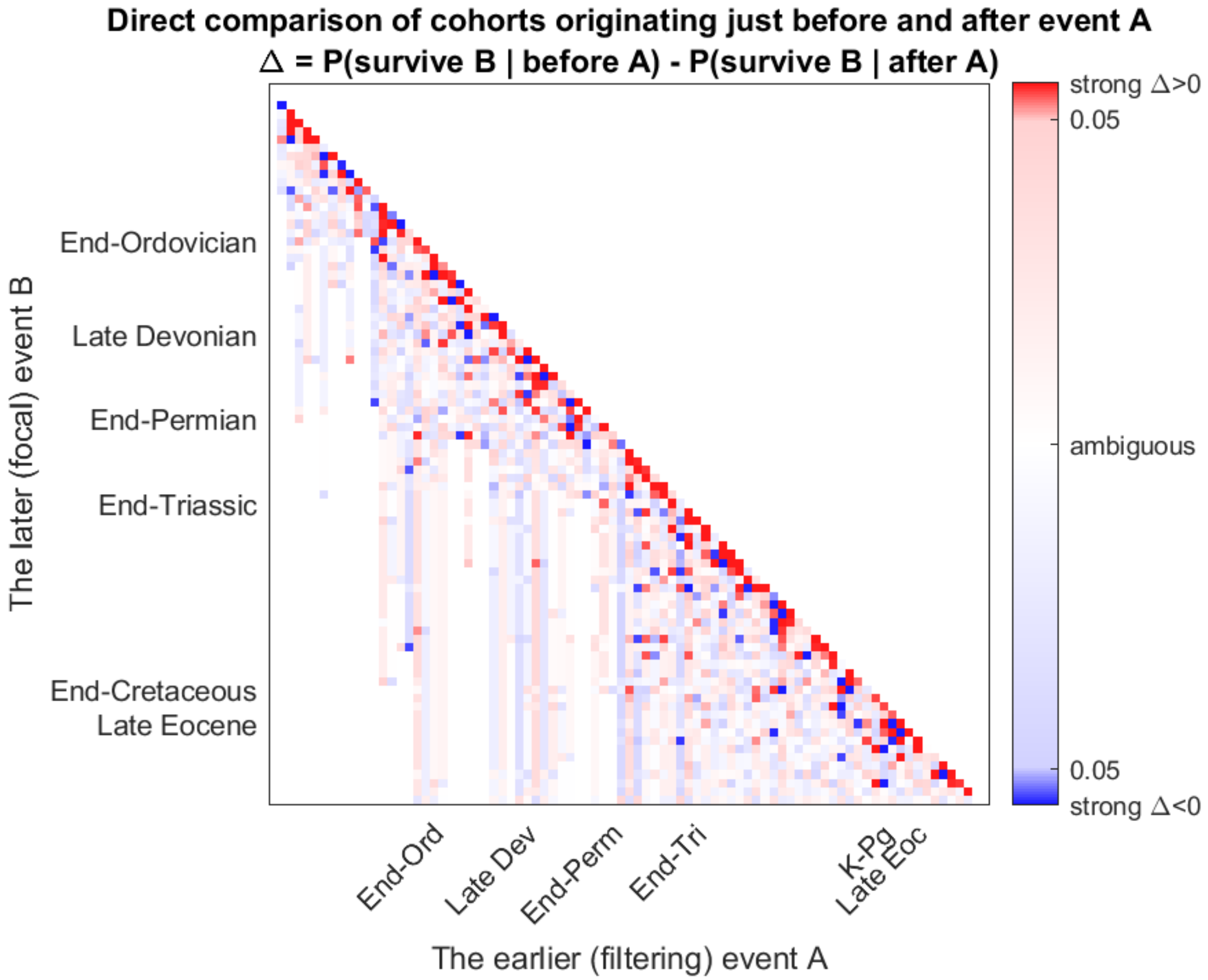

FIG. S1. **Direct sister-cohort comparisons provide limited statistical power.** Each pixel compares the survival probability through a later (focal) extinction event B of genera originating immediately before and immediately after an earlier (filtering) event A, conditioned on being alive immediately before B. Events A and B run through all 84 epoch boundaries recorded in the dataset; the ticks on the axes mark the Big Five extinction events and the Late Eocene event. Cell color indicates the posterior evidence for the sign of Δ, where $\Delta > 0$ corresponds to greater survival of the older (pre-A) cohort. Gray cells indicate comparisons for which one or both conditioned cohorts are empty. Although the matrix shows an overall bias toward positive Δ, the evidence is generally weak and becomes sparse away from the diagonal because of progressive cohort attrition.

bioRxiv preprint doi: https://doi.org/10.64898/2026.07.21.739780; this version posted July 24, 2026. The copyright holder for this preprint (which was not certified by peer review) is the author/funder, who has granted bioRxiv a license to display the preprint in perpetuity. It is made available under a CC-BY 4.0 International license.

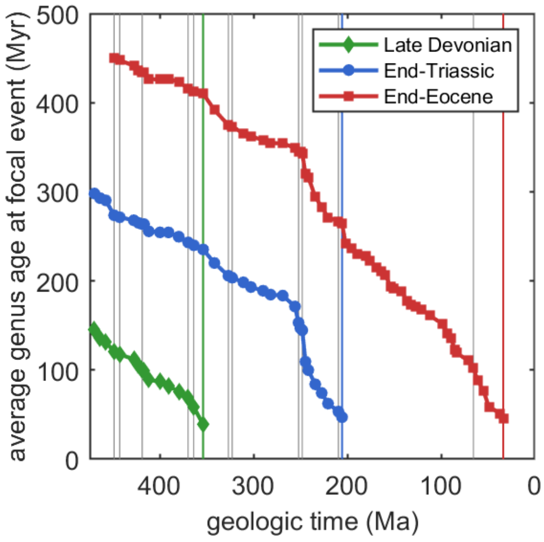

FIG. S2. **Mean cohort age changes smoothly across earlier extinction events.** For the same three focal extinction events shown in Fig. 1C, each curve gives the mean genus age at the focal extinction event among genera contributing to the corresponding point on the event survival curve. Figure layout is identical to Fig. 1C to facilitate visual comparison: gray vertical lines mark earlier extinction events with extinction fractions exceeding 40%, and colored vertical lines mark the focal events. Although major extinction events alter the composition of the retrospective cohorts, the resulting changes in mean cohort age are comparatively smooth. Thus, the sharp discontinuities observed in the event survival curves of Fig. 1C cannot be explained solely by changes in cohort age.

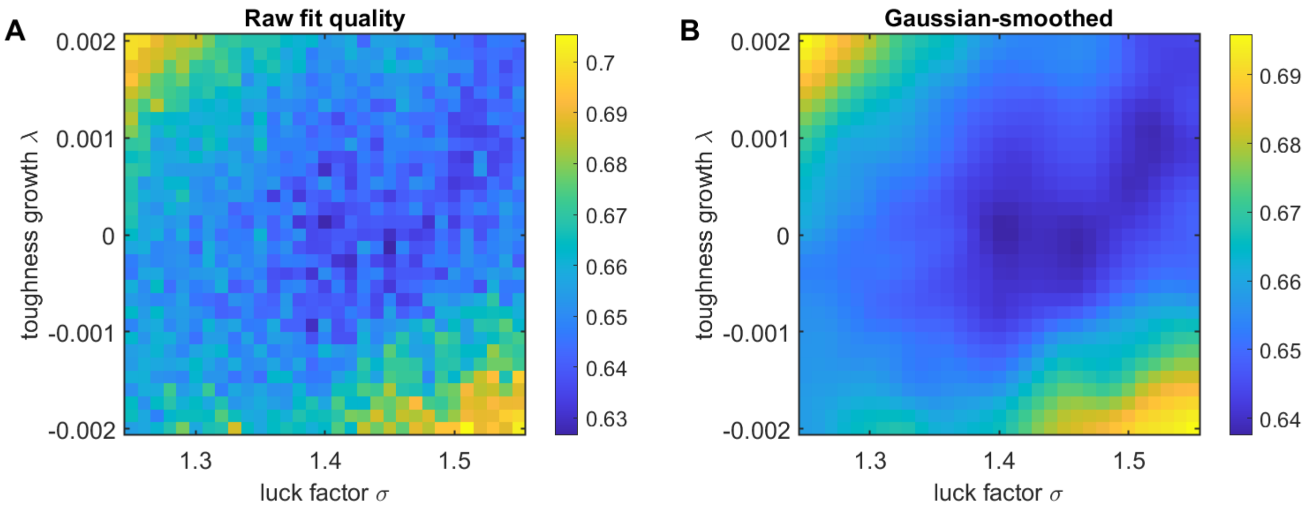

FIG. S3. **Local refinement of the luck-factor and toughness-growth fit. A:** Full-curve z-RMS fit error evaluated on a 31×31 refined parameter grid centered on the minimum of the coarse grid shown in Fig. 4. Each grid point is averaged over the same 10 synthetic model replicates used in the coarse sweep. **B:** The same refined-grid fit surface after modest Gaussian smoothing, shown to highlight the underlying low-error region.

bioRxiv preprint doi: https://doi.org/10.64898/2026.07.21.739780; this version posted July 24, 2026. The copyright holder for this preprint (which was not certified by peer review) is the author/funder, who has granted bioRxiv a license to display the preprint in perpetuity. It is made available under a CC-BY 4.0 International license.

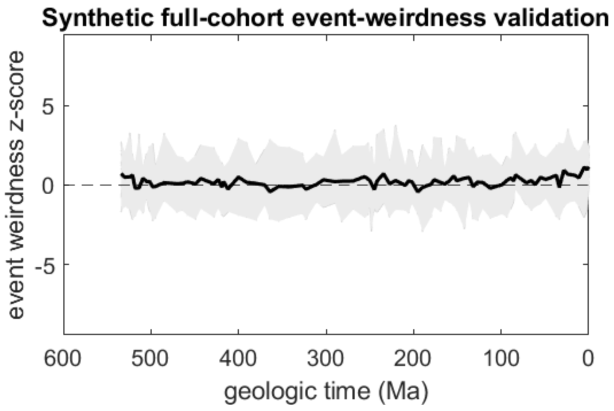

FIG. S4. **Synthetic validation of the event-weirdness statistic.** Event weirdness scores computed from synthetic fossil records generated by the fitted toughness-plus-luck model. The black curve shows the median, and gray band the range of the weirdness score computed across 20 synthetic replicates. The y-axis scale is identical to that of Fig. 5 in the main text. As expected under the null model, synthetic event weirdness fluctuates around zero, with no extinction event consistently exhibiting unusually large positive or negative scores.

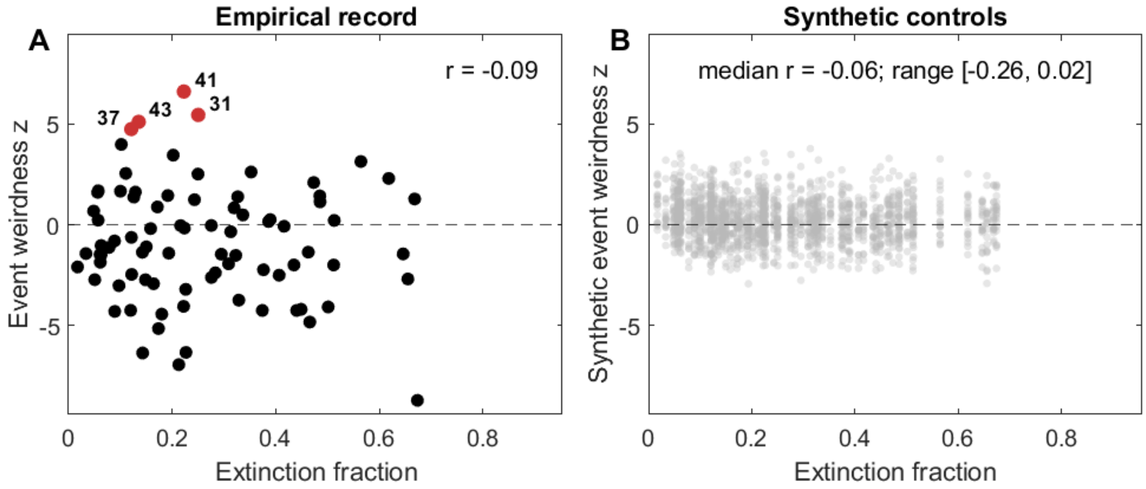

FIG. S5. **Event weirdness is independent of extinction magnitude.** (A) Event weirdness score versus extinction fraction for the empirical Sepkoski record. The four unusually positive events highlighted in Fig. 5 (event indices 31, 37, 41, and 43) are shown in red. The weak correlation (Pearson $r = -0.09$) indicates that event weirdness is not simply a consequence of extinction magnitude. (B) The corresponding analysis for synthetic fossil records generated by the fitted toughness-plus-luck model. Each gray point is one event from one synthetic replicate; all replicates share the empirical extinction fractions by construction. Correlations remain weak, confirming that the event weirdness score is effectively decoupled from event magnitude.
